# Electron-blocking by the potential barrier originated from the asymmetrical local density of state in the oxide semiconductor

**DOI:** 10.1038/s41598-017-18420-9

**Published:** 2017-12-21

**Authors:** Hyeon-Jun Lee, Katsumi Abe, Jun Seo Kim, Myoung-Jae Lee

**Affiliations:** 1 Intelligent Devices & Systems Research Group, Institute of Convergence, DGIST, Daegu, 42988 Korea; 2 Global Center for Bio-Convergence Spin System, DGIST, Daegu, 42988 Korea; 3 Silvaco Japan Co., Ltd., Nakagyo-ku, Kyoto 604-8152 Japan

## Abstract

Defect generation in oxide semiconductor thin-film transistors under high-voltage driving has not been studied in depth despite being a crucial bottleneck in the making of the integrated circuit utilized in an oxide semiconductor. Here we report on the origin of the asymmetrical transport characteristics caused by the degradation in the oxide semiconductor during integrated circuit driving. The variation of the current profiles based on test conditions is related to the generation of local defect states in the oxide material; this generation could be caused by the structural change of the material. The numerical calculations show that the flow of the electron is blocked by the “electrical pocket” formed by the electric-field distortion due to the local defect states near the edge of the electrode.

## Introduction

Metal-oxide semiconductors including those of zinc oxide have become key materials for large-area and flexible electronics because the uniformity of the electrical properties is guaranteed by the wide coverage of the delocalized s and 5s electrons in the material containing the heavy element^1^. Therefore, the interest in metal-oxide semiconductors is increasing in industries^2^ and academia^3,4^. Other noteworthy features of oxide semiconductors include structural disorder and defects^5^. One of the well-known defects is the “oxygen-vacancy”^6,7^ in the crystalline oxide semiconductor; this significantly affects the corresponding electrical transport properties^8^ as well as the optical characterictics^6^. Moreover, some of the defects are used as tools to enhance electrical characteristics, such as hydrogen doping^9^, and serve as an impediment to device driving, for example, deep states formed by oxygen-vacancies^10^.

The generation of the defects in an oxide semiconductor is largely determined by stoichiometry^11^, growth temperature/environments^12,13^, system energy^14^, and various other causes^2,10,15^. In addition, defect formation is dominated by electric driving conditions or the material environment. So far, most studies have been limited to state generations caused by the fixed charge^16,17^ at the interface between gate insulator and active material, or to the state generation in the bulk or interface. In our previous study^18^ conducted on the defect formation during electrical driving in oxide semiconductor devices for integrated circuit (IC) applications, “asymmetrical local defect states (ALDS)” were reported during the IC driving in the oxide channel area; this could be a stumbling block in the application of oxide semiconductors. Although few studies^18–20^ have been conducted on the ALDS in a semiconductor-based thin film transistor (TFT), our understanding of the ALDS remains insufficient because no study has been conducted on the local defect state as a function of active layer positioning and its affect in terms of electrical properties. Therefore, the correlation of the electric-field and carrier-concentration distributions with the various positioned local defect states should be studied to clarify where the asymmetrical electrical properties are induced in an oxide semiconductor.

Here, we investigated the physical properties as a function of the ALDS positioning in the active channel layer. In the testing, we employed amorphous indium gallium zinc oxide (*a*-IGZO) as the channel material for n-type oxide transistors fabricated on a gate insulator. To detect the defect state indirectly, the drain current–gate voltage (*I*_*d*_ − *V*_*g*_) curves and current reduction were measured in the samples forming the local defects due to pulse-type drain stress at room temperature. The local defect states were positioned from source to drain electrodes to understand the effect of the local defects through a technology computer aided design (TCAD) simulation. Depending on the local defect position, the transport profile between source and drain exhibited different behavior and current reduction.

In this study, we quantified the electron-blocking phenomena by analyzing the current reduction mechanism of the device after exposure to the pulse-type drain stress. The local increase of the acceptor-like Gaussian defect state from 1.5 × 10^17^ to 6.0 × 10^17^ cm^−3^ eV^−1^ resulted in current reduction around the threshold voltage. The ALDS formed by the pulse-type high-voltage drain stress (HVDS) disturbed the electron modulation when the gate voltage swept under the reverse source/drain. The electric field at the edge of the source electrode was abnormally reduced, and the magnitude was approximately one order less than that in the normal transistor. The electric-field pocket causes the obstacle to inject an electron from the source electrode, and the electron transport was confirmed to be improved by the shift of the electric-field pocket. To the best of our knowledge, this is the first study to report that the electron transport is impeded by the electric-field pocket caused by ALDS.

## Results and Discussion

### Current reduction by the asymmetrical local defect states

**Figure 1:**
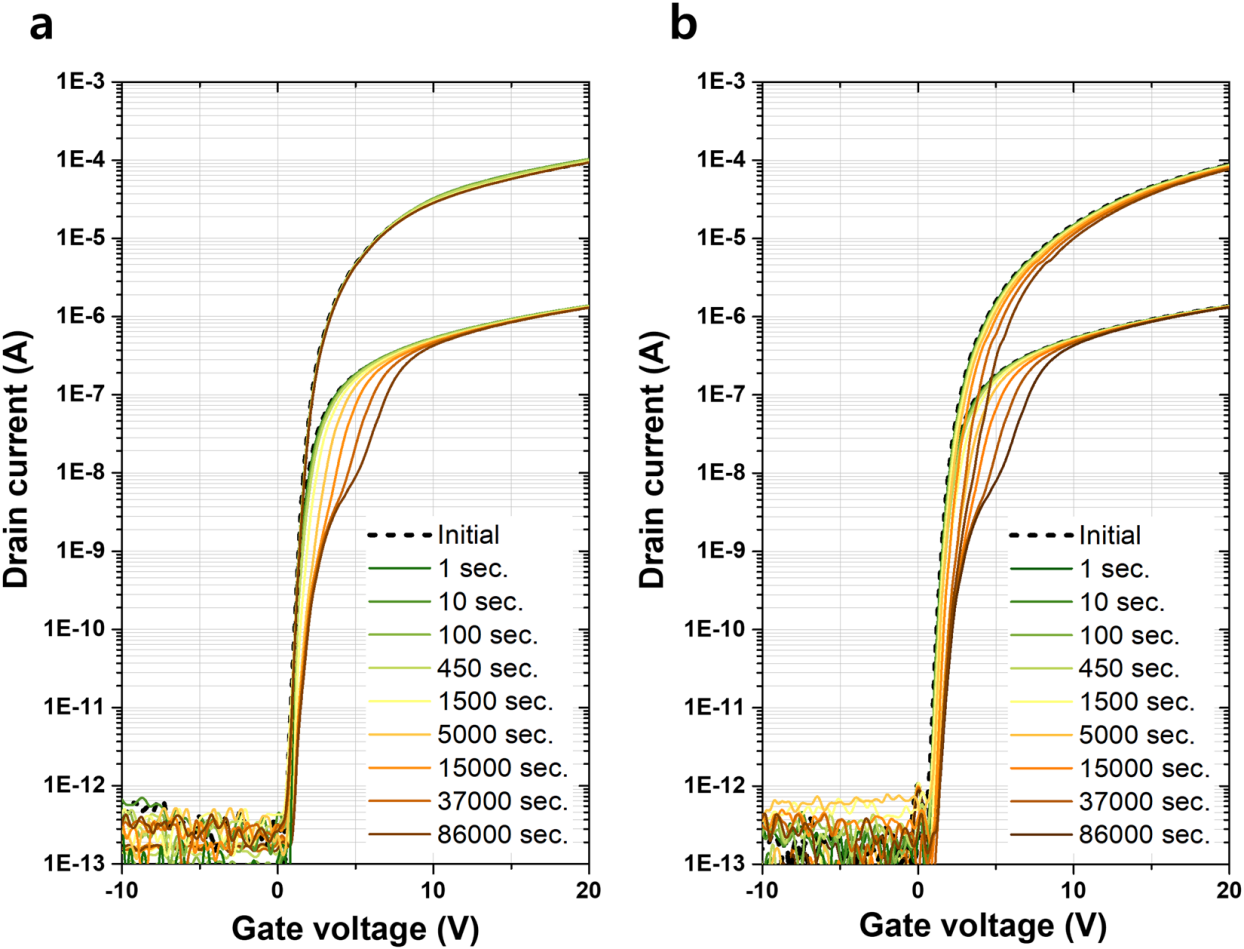
Current lowering at the low gate field. *I*_*d*_ − *V*_*g*_ characteristics as a function of the pulse-type high-voltage drain stress time. The voltage *V*_*ds*_ of 10 and 0.1 V was applied for *I*_*d*_ − *V*_*g*_ sensing. (**a**) *I*_*d*_ − *V*_*g*_ forward sweep measurement (“forward” implies the equivalent source/drain direction of the stress with the sensing). (**b**) *I*_*d*_ − *V*_*g*_ reverse sweep measurement (“reverse” implies the different source/drain direction of the stress with the sensing). For the *I*_*d*_ − *V*_*g*_ sweep for sensing, the applied high voltage at the source electrode under the drain is grounded.

Figure 1a and b show the electron transfer characteristics depending on the pulse-type HVDS time [1 kHz frequency, duty cycle 1%, *t*_on_ = 1 μs, turn on voltage (*V*_*ds*_) = +40 V, and turn off voltage (*V*_*g*_) = 0 V] for forward and reverse sensing, respectively. A current drop and small positive shift can be observed in the *I*_*d*_ − *V*_*g*_ curves in both the directions at *V*_*ds*_ = 0.1 V. However, the *I*_*d*_ − *V*_*g*_ curves at *V*_*ds*_ = 10 V show different characteristics for the forward and reverse direction measurements. Current reduction or change based on the pulse-type HVDS time is not observed in the forward *I*_*d*_ − *V*_*g*_ curve, and after a stress of 86 ks, the current profile shows an equivalent value and trend compared with the initial *I*_*d*_ − *V*_*g*_ characteristic, as shown in Fig. 1a. In contrast, the reverse *I*_*d*_ − *V*_*g*_ curve at *V*_*ds*_ = 10 V after the stress shows a serious current drop at the low gate voltage region and is clearly distinguished from the initial curve. These asymmetrical characteristics in the transfer curves at *V*_*ds*_ = 10 V were similar to those observed in Si-based TFTs under DC stress and explained through the deep state generation^21^. A small positive shift was observed to originate from either the negative fixed charges, generated by the high positive drain voltage at the interface between the gate insulator (GI) and active layer, or the generation of the deeper acceptor-like defects^2^.

**Figure 2:**
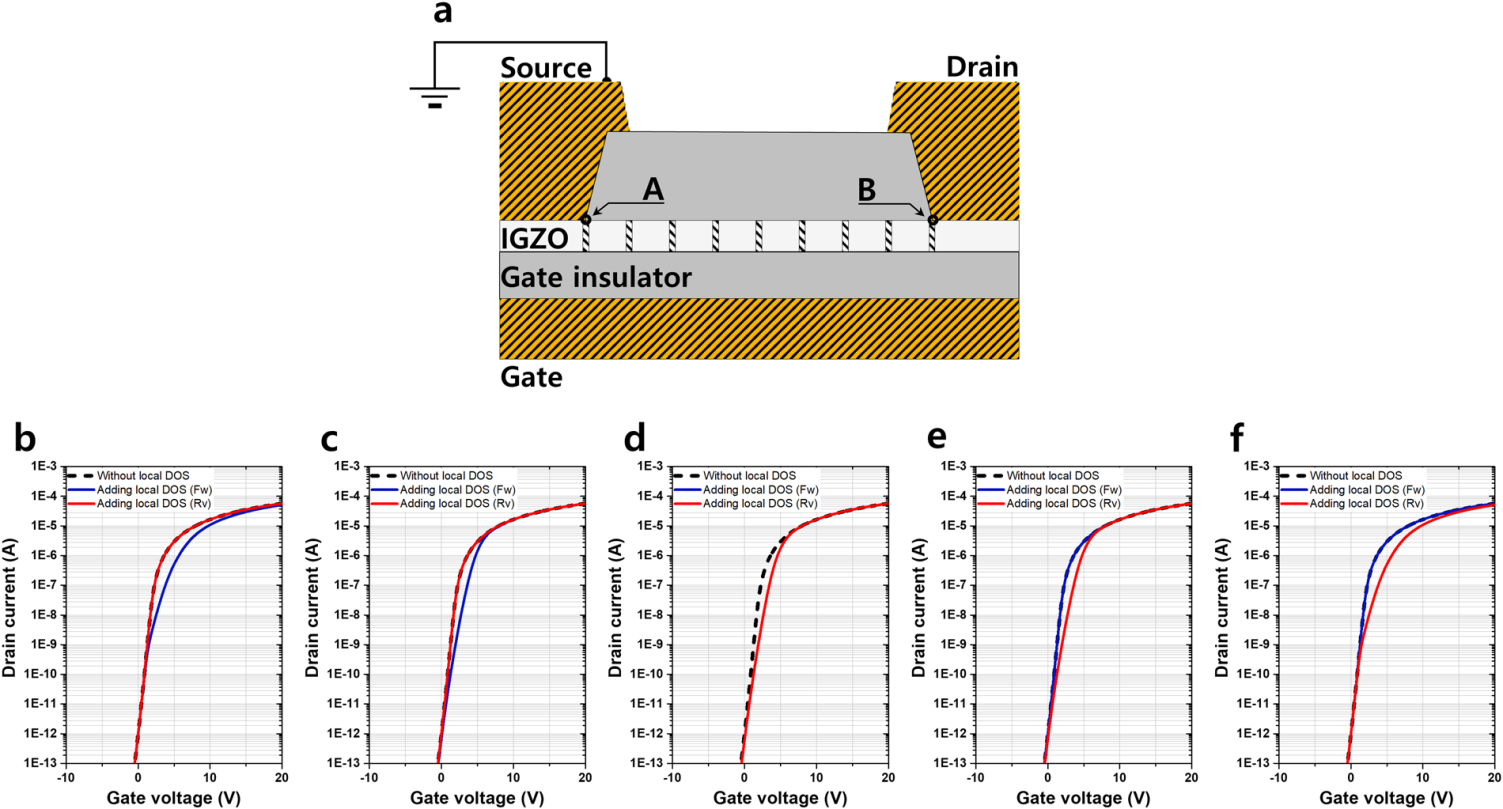
Asymmetric local defects states and electric transport. (**a**) Cross-sectional schematic image of an oxide semiconductor TFT (etch stopper type). The left and right electrodes are the source and drain, respectively, and the two points “A” and “B” indicate the edges of the source and drain, respectively. The local defect states are positioned at nine local regions (0, 1, 2, 3, 4, 5, 6, 7, and 8 μm). The width of the local defect is 0.4 μm and the local density of state (DOS) is 6.0 × 10^17^ cm^−3^ eV^−1^ for the acceptor-like Gaussian state below CBM. (**b–f**) *I*_*d*_ − *V*_*g*_ characteristics for defects positioned at (**b**) 0, (**c**) 1, (**d**) 4, (**e**) 7, and (**f**) 8 μm obtained using TCAD ATLAS numerical simulation. The dashed line represents the *I*_*d*_ − *V*_*g*_ characteristics without a local defect (acceptor-like Gaussian state of 1.5 × 10^17^ cm^−3^ eV^−1^ was applied). The blue and red solid lines show the forward (Fw) and reverse (Rv) source/drain sweep measurements, respectively. The blue and red curves are overlapping as shown in (**d)**.

To understand the asymmetrical *I*_*d*_ − *V*_*g*_ curve in Fig. 1, a 2D numerical TCAD simulation was used to simulate the device characteristics. Figure 2a shows the cross-sectional model of the device after pulse-type HVDS. The simulated structure has a 40 nm-thick channel with a length of 8 μm, including the 2 μm overlapping regions below the source and drain electrodes. The acceptor-like Gaussian local defect states with peak density, energy center, and energy width of 6.0 × 10^17^ cm^−3^ eV^−1^, 0 eV, and 0.5 eV, respectively, were positioned at nine areas (0, 1, 2, 3, 4, 5, 6, 7, and 8 μm from the source edge) and the width of each area was considered to be 0.4 μm. The peak densities of acceptor- and donor-like Gaussian states near the conduction band minimum (CBM) were estimated as 1.5 × 10^17^ and 1.3 × 10^17^ cm^−3^ eV^−1^, respectively, without the application of the pulse-type HVDS. The local defect state is defined as the acceptor-like Gaussian state below the CBM because it seems to be generated by the breaking of the weak oxygen bonds caused by the energy transfer from the hot electron of the high voltage pulse stress. However, further study is necessary to ensure the breaking of the weak oxygen bonds. Figure 2b–f show the simulation results with different local acceptor-like Gaussian defect positions for both direction sweeps, that is, source/drain forward and reverse direction sweeps at *V*_*ds*_ = 10 V. In the case of the local defect state at 0 μm (position A; the edge of source electrode), the forward measurement shows current value and profile equivalent compared with those of the reference sample (without the pulse-type HVDS). However, if the potential direction is changed, that is, in the case of measurement sweep direction, current reduction is observed by the acceptor-like local defect state at 0 μm (position A). Figure 2b clearly shows the current drop at the low gate voltage of less than 10 V. In the case of the local defect at 1 μm, Fig. 2c shows that the current gap between the forward and reverse sweeps is smaller. The drain current at the reverse sweep is still equivalent with that of the reference sample (without experiencing HVDS). Figure 2d shows the *I*_*d*_ − *V*_*g*_ characteristics of the local defect state positioned at the center of the channel length, that is, at 4 μm. The current profiles of both the forward and reverse sweeps overlap, and a small current drop is observed compared with that of the reference sample. The current profiles of the local defect state positioned at 7 and 8 μm are shown in Fig. 2e and f, respectively. When the HVDS was applied at the drain side in Fig. 2a, the local defect states were generated at position B. Fig. 2f matches well with the measured data in Fig. 1. In the forward *I*_*d*_ − *V*_*g*_ measurement, no current change is observed even if the acceptor-like Gaussian state as the local defect state is at position B, that is, 8 μm, which is four times larger than the defect in the reference sample. The current reduction is only observed in the opposite sweep direction. In the applied lower lateral E-field (*V*_*ds*_ = 0.1 V sensing), a symmetrical current reduction is observed wherever the potential barrier is placed because the potential barrier due to the defects is higher than the electrical potential difference induced by *V*_*ds*_.

### Abnormal electric field distortion by the local defect states

**Figure 3:**
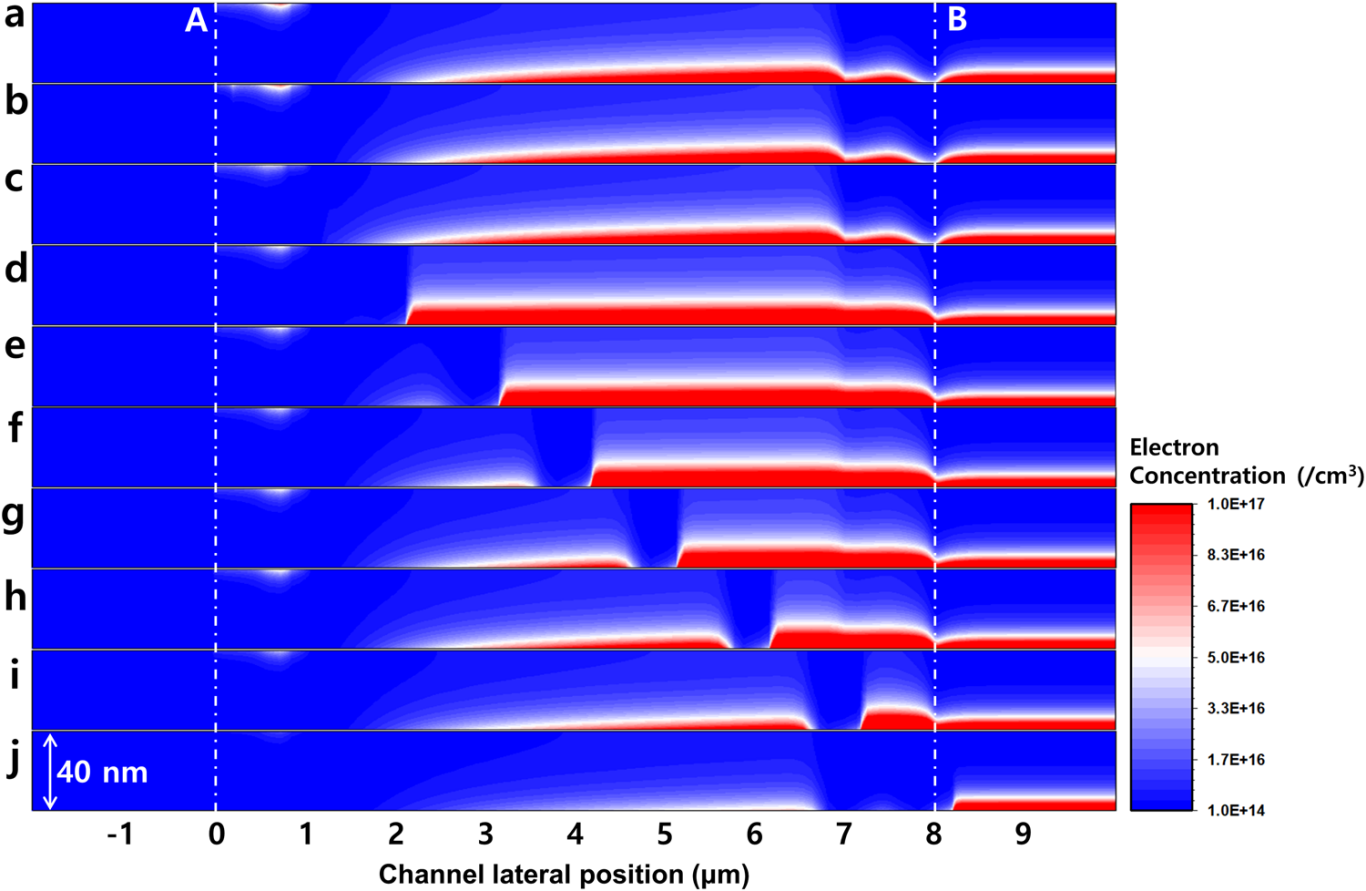
Electron concentration as a function of local defect state position. The electron concentration distribution of the oxide semiconductor cross-sectional channel region under a gate voltage of 5 V, source electrode of 10 V, and drain electrode of 0 V (reverse sweep measurement). Points “A” and “B” at 0 and 8 μm, respectively, are equivalent positions to points A and B shown in Fig. 2a. The height of each graph represents the thickness of the sample, i.e., 40 nm. (**a**) Electron concentration of the channel region at the low gate voltage (5 V) without the local defect states. (**b**–**j**) The distribution of the electron concentration as function of the local defect position at (**b**) 0, (**c**) 1, (**d**) 2, (**e**) 3, (**f**) 4, (**g**) 5, (**h**) 6, (**i**) 7, and (**j**) 8 μm.

It was confirmed that the asymmetrical *I*_*d*_ − *V*_*g*_ characteristic originated from the acceptor-like Gaussian defect state below the CBM through the TCAD simulation. To verify the electronic modulation under the driving, the distribution of the electron concentration was numerically calculated as a function of the local defect position. Figure 3a shows the modulated electron concentration of the reverse sweep measurement under the 5 V gate and the 10 V drain in the reference sample (none experienced the HVDS). Hence, points A and B are the edges of the drain and source electrodes, respectively (reverse measurement). The electron concentration was observed to be more than 1.0 × 10^17^/cm^3^ at the bottom of channel layer at the 5 V gate. This concentration shows the highest density at 7 μm, forms a tail, and reduces as moving toward point A because of the electric field contribution. In addition, a kink is observed for the electron concentration at 7 μm, and seems to be related with the etch stopper layer SiO_x_, thus disturbing the electric field. In the case of the reverse sweep measurement, the local defect being positioned at 0 and 1 μm does not disturb the electron modulation at the reverse measurement, as shown in Fig. 3b and c. The distribution of the modulated electron begins to change from 2 μm (Fig. 3d) of the local defect state. The electron concentrations of the sample with local defects at 2, 3, 4, 5, 6, and 7 μm clearly show the electron distributions in Fig. 3d–i. At the local defect point, the modulated electron concentration is reduced more than 1 order of magnitude. Figure 3j shows the result of the electron concentration of *a*-IGZO channel layer containing the local defect state positioned at 8 μm. The concentration is rarely distributed in the entire channel area even though the gate electric field applied is 5 V. Especially, there exists an extremely low electron concentration at the edge of the electrode at point B. These low electron concentrations give rise to asymmetrical abnormalities in the reverse electrical measurement, as shown in Fig. 1.

**Figure 4:**
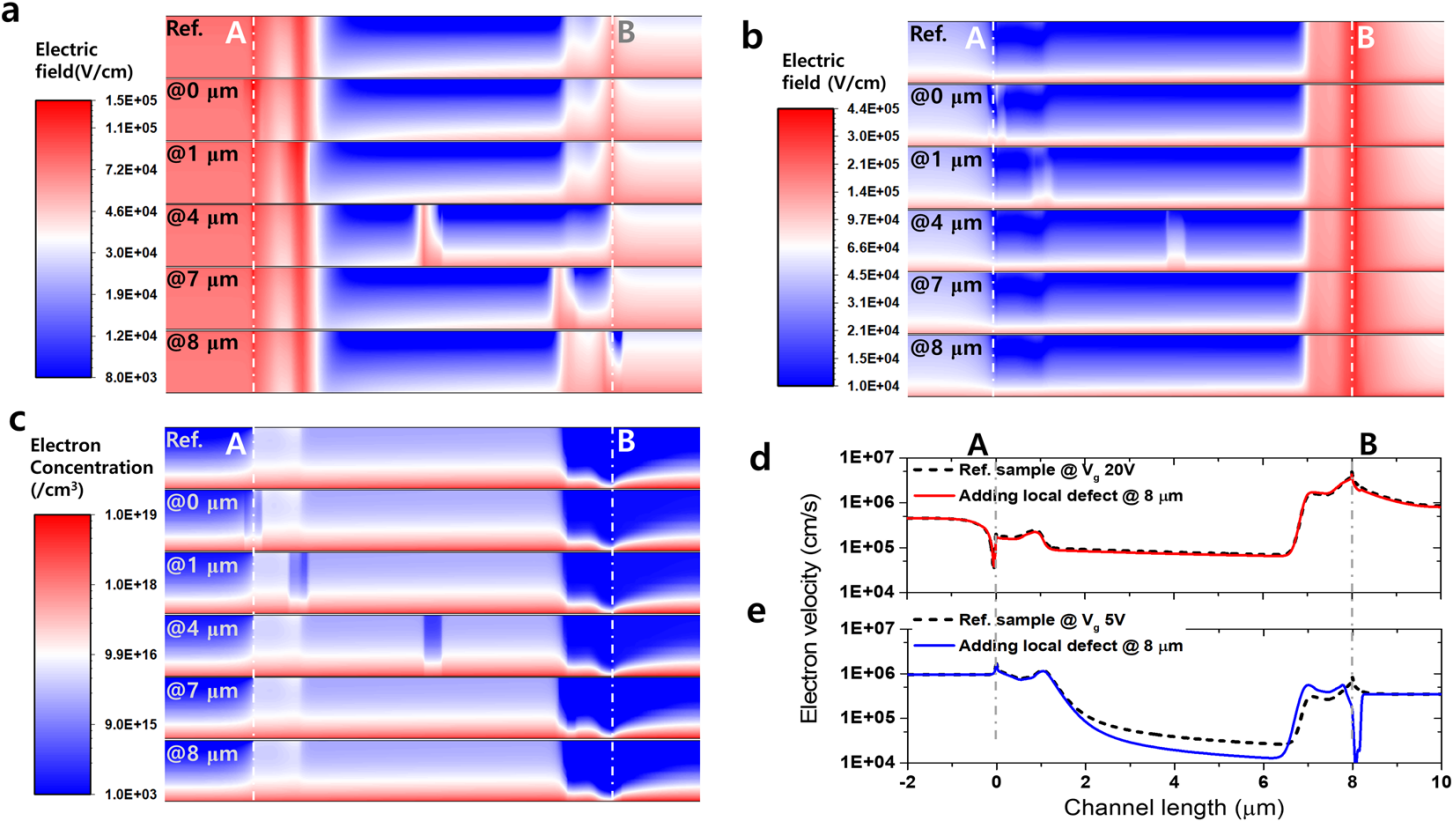
Simulation of physical properties as local defects. The electric field, electron concentration, and electron velocity as calculated using the ATLAS 2D simulation. The source and drain were fixed at 10 and 0 V, respectively (reverse sweep measurement). Points “A” and “B” are set at 0 and 8 μm, respectively, which are equivalent to the position of points A and B in Fig. 2a. Electric field distribution under a gate voltage of **(a)** 5 V and (**b**) 20 V. (**c**) Simulated electron concentration (under *V*_*ds*_ = 10 V and *V*_*g*_ = 20 V) in the oxide semiconductor channel region. The electron velocities under the electric field were calculated based on the applied gate voltage. (**d**) For a high gate voltage of 20 V, the dashed line represents the reference sample (without local defect state) and the red solid line indicates the local defect state positioned at 8 μm. (**e**) For a low gate voltage of 5 V, the black dashed and blue solid lines represent the velocity profiles of the reference sample and the sample with the local defect states positioned at 8 μm, respectively.

These asymmetrical electron distributions are caused by the distortion of the electric field. Figure 4a shows the absolute-value distribution of the total electric field without and with the local defect states at 0, 1, 4, 7, and 8 μm for the reverse source/drain sweep at *V*_*gs*_ = 5 V and *V*_*ds*_ = 10 V. The electric field distribution of the reference sample (without the HVDS) shows the smooth changing of the values from 10^5^ to 10^4^ V/cm. The electric field is at a lower value at point B than at point A because of the consideration of the reverse source/drain sweep measurement; the electrode at point B was set to 0 V and that at point A was set to 10 V as drain. The distortion of the electric field caused by the local defect states being positioned at 0 and 1 μm is not clearly shown because the intrinsic electric field is extremely strong at the drain side near point A. Owing to the acceptor-like Gaussian defects, the electric field could be enhanced at the local defect position, as shown at 4, 7, and 8 μm in Fig. 4a. In the case of the local defect located at 8 μm, the reduction in the electric field was observed at the local defect position, that is, point B. In contrast, the distribution of the electric field at the gate voltage of 20 V showed the opposite electric field trend compared with the gate voltage of 5 V, as shown in Fig. 4b. A gate voltage of 20 V, which is higher than the drain (10 V at the point A), was applied; this created a higher electric field at point B. The electric fields of the local defects at 0, 1, and 4 μm showed a behavior similar to that of an increasing electric field; however, the electric field at the local defect near point A was different from that at the low gate voltage of 5 V. The electric field of the local defect state at 8 μm showed very similar behavior with the field distribution of the reference sample (without the HVDS), giving rise to very similar transport properties at the high gate voltage. These similarities were confirmed at the electron concentration under the application of high gate voltage, as shown in Fig. 4c. Figure 4d and e plot the electron velocities at the top of *a*-IGZO channel layer with respect to the local defect at 8 μm under low (5 V) and high (20 V) gate voltages, respectively. The dashed lines indicate the reference sample in both graphs. As discussed earlier, at the high gate voltage (20 V), no difference was observed between the reference sample and the sample with local defect sates. However, at the low gate voltage (5 V), the electron velocity showed a large difference, especially at 8 μm.

### Electric field pocket as potential barrier

**Figure 5:**
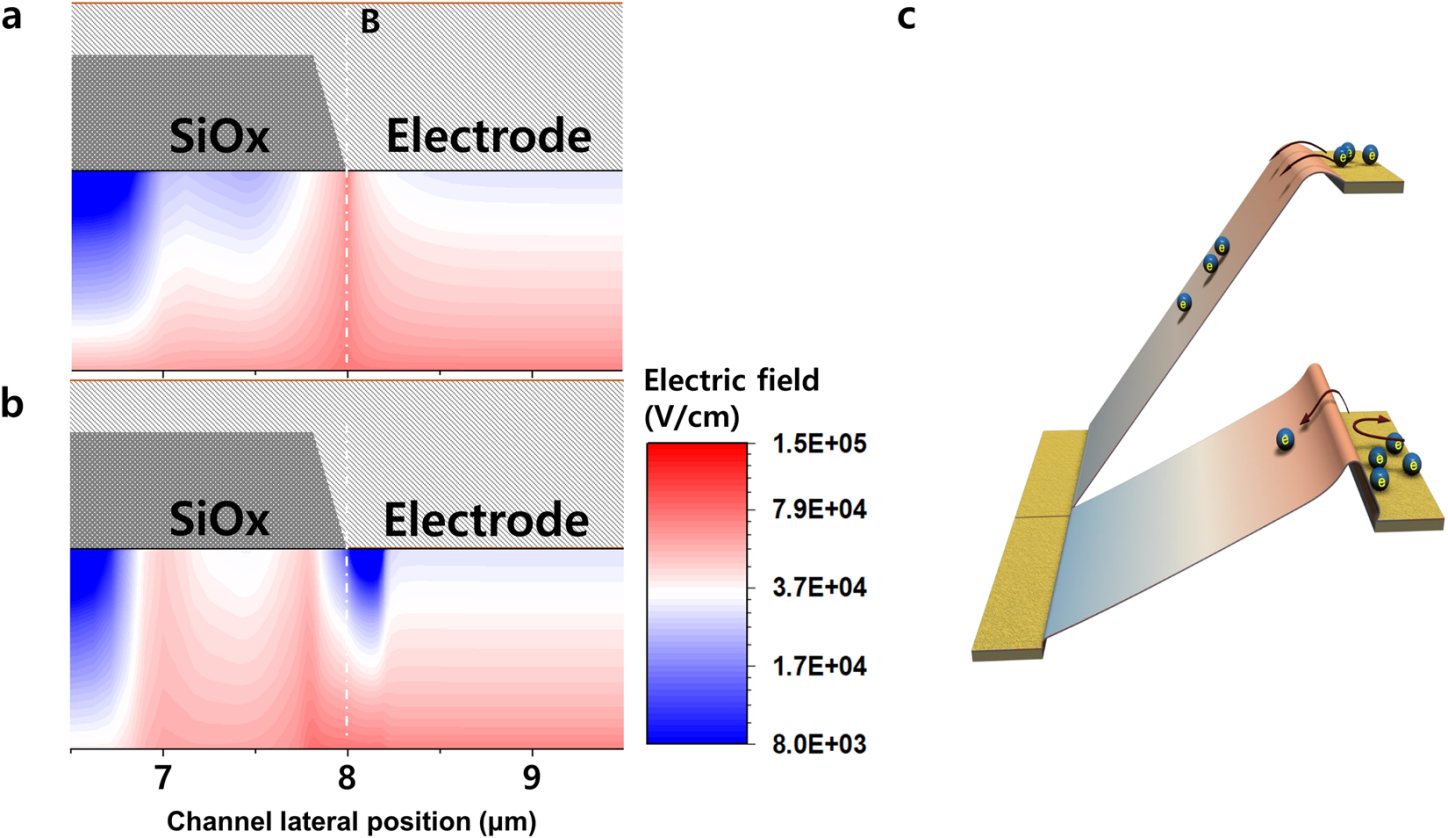
Electric field pocket as a potential barrier. (**a**) Electric field distribution near point “B” region between 6.5 and 9.5 μm for the reference sample (without the local defect states). (**b**) Electric field distribution near the point “B” region for the sample positioned at 8 μm (7.8–8.2 μm, the width of the local defect states is 0.4 μm). Point “B” at 8 μm is equivalent to the position of point B in Fig. 2a. The electric-field pocket is observed at point B, as represented by the deep blue (low electric field) region. (**c**) Schematic image of the potential barrier.

Figure 5a and b show the local electric field distribution [under the low gate voltage (5 V)] at the edge of the electrode, that is, point B [the voltage of the electrode points B and A was 0 and 10 V at the gate of 5 V, respectively (reverse source/drain sweep)], for the reference sample and the sample with the acceptor-like Gaussian local defect states at 8 μm. For the reference sample without the local defect state, the electrons from source electrode flowed through the channel top through the bulk *a*-IGZO and reached the bottom of the active layer. Figure 5a shows that the electric field at point B naturally increases and spreads out to the bottom, and the electron injected from the electrode (source) is accelerated under the electric field gradient. This represents normal electronic transport in the transistor. However, in the case of *a*-IGZO transistor with the local defect state at point B, the electric field was distorted, as shown in Fig. 5b. At point B, the electric field shows crucially low values (electric-field pocket) less than 10^4^ V/cm, which is less than one order of magnitude lower than the value in the reference sample. The electron should be injected from the electrode (source) through the edge of the electrode at point B, whereas the flow of the electron was blocked by the “electric-field pocket,” which was a potential barrier at point B. The electron was unable to reach the bottom of *a*-IGZO because of the electron being blocked at the bottleneck, that is, point B. These electrons being blocked by potential barrier at the low gate voltage region reduce current in the *I*_*d*_ − *V*_*g*_ transfer curve (Fig. 5c).

**Figure 6:**
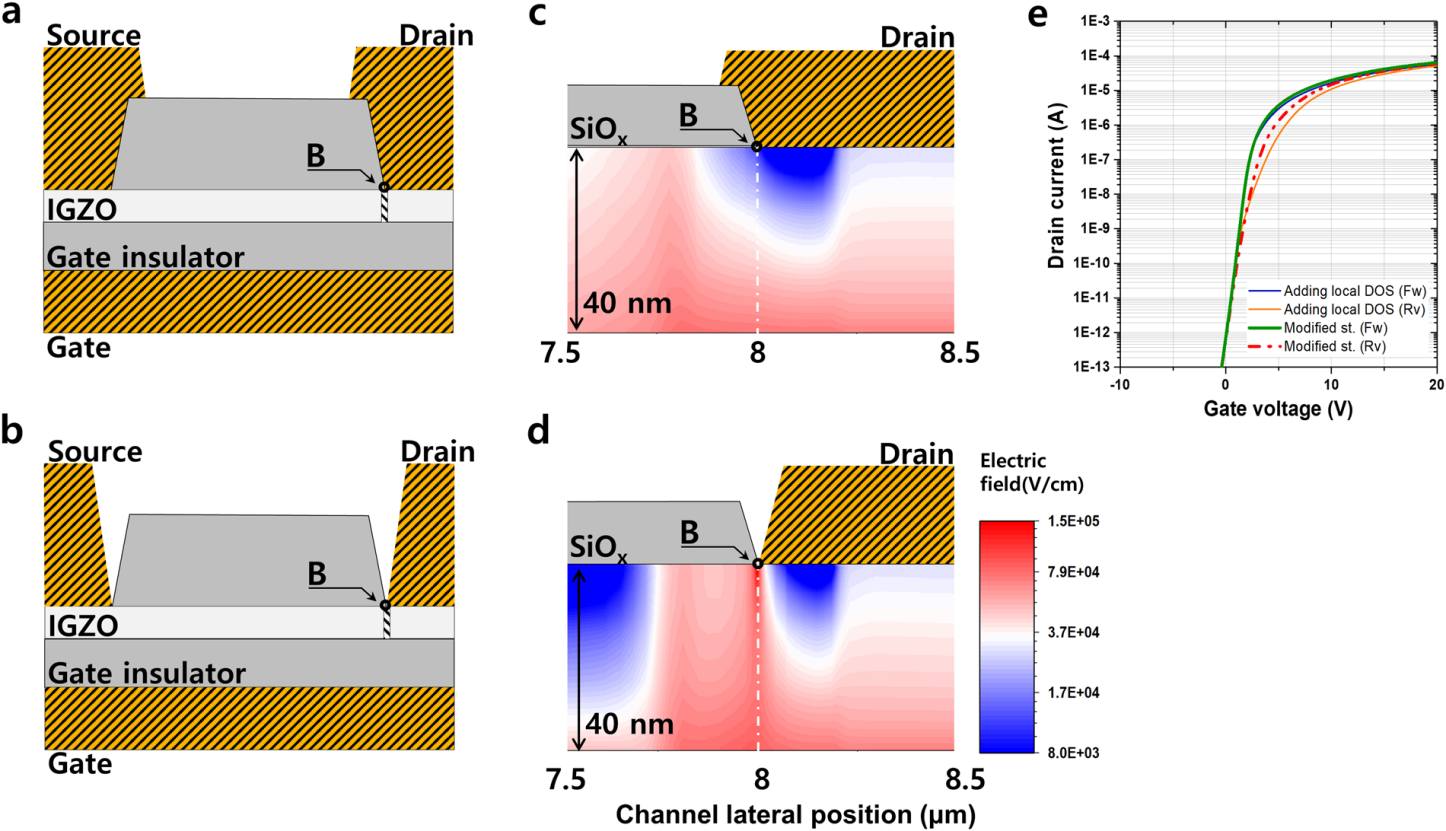
Electric field distribution as electrode position. The source/drain electrode position is changed from (**a**) on the etch stopping SiO_x_ layer to (**b**) the end of the SiO_x_. Let the local defect be at position “B”. (**c**) The electric field distribution in the conventional etch stopper TFT structure as shown in (**a)**, and (**d**) the electric field distribution of the oxide semiconductor channel region in the modified source/drain metal position as shown in **b**. (**e**) *I*_*d*_ − *V*_*g*_ plot shows both direction sweep simulation data as a function of the conventional TFT structure with the local defect states [blue solid line (forward sweep) and orange solid line (reverse sweep)] and of the modified structure with the local defect states [green solid line (forward sweep) and red dashed line (reverse sweep)].

To clarify the electric field contribution of the electron transport, the field distributions were calculated using the ATLAS 2D simulator. Under the fixed TFT structure, the source/drain electrode position was changed from on etch stopping SiOx layer to the end of the SiOx, as shown in Fig. 6a and b. The local defect state (the peak density is 6.0 × 10^17^ cm^−3^ eV^−1^) and the position (B point) are equivalent in the both structures. The shift of the source/drain electrode position changes the electric field distribution at 8 μm in channel. As shown in Fig. 6c and d, the electric field pocket is observed in both structures but the electric field is observed at 8 μm (being positioned defect) and shows different behavior. Unlike the electric field in the blue region, the order of 10^4^ V/cm at 8 μm position in Fig. 6c, the field strength in the Fig. 6d is approaching 10^5^ V/cm order (red region). By shifting the electric field pocket, the electron is able to inject more easily from electrode to oxide semiconductor channel compared with the conventional semiconductors. The current reduction at the low gate field is plainly improved by the detouring of the electric field pocket, as shown in Fig. 6e.

## Discussion

In this paper, we report on the origin of asymmetrical transport characteristics in oxide semiconductor TFTs in an IC driver. The variation in current profiles based on test conditions is related to local defect states in the channel. The local defect states formed at the edge of the electrode distort the electric field distribution with an increase in the density of states. The local defect states formed by the chain reaction of hot electron energy transfer^18^ in IC drivers hinder the flow of electrons by forming an electric-field pocket. In addition, the acceptor-like Gaussian local defect states capture electrons, decrease the Fermi level, and form an electrical-potential barrier around the surrounding area. When the local defect area is positioned at the source, the potential barrier influences the potential distribution and decreases the electric field from the gate or drain to the source. Thus, the defects generate an “electric-field pocket,” resulting in a drop in the current in the *I*_*d*_ − *V*_*g*_ transfer curve under low gate voltage. If the gate voltage is high, the defect states are filled with electrons and the potential barrier decreases. The current drop then disappears but grows continuously while the device is driven.

Since the development of the oxide semiconductor, extensive research has been conducted on the threshold voltage shift caused by gate charging and state generation on the interface of the gate insulator. However, research on alternating driving signals for IC development or the application of the gate driver in electronic devices based on oxide semiconductors is restricted, and in-depth studies are currently necessary. The phenomenon of easy bond breaking originating from an ionic bond structure under high voltage driving, which is the inherent problem of oxide semiconductors, was presented, and the cause of this problem was analyzed in this study. The results of this study are expected to serve as a basis for further research aiming to improve the instability of the oxide semiconductor for IC drivers.

## Methods

### Device Fabrication

A molybdenum (Mo) gate electrode (150 nm) was formed on an amorphous SiO_x_/Si substrate and a 200 nm-thick SiO_x_ single layer was then deposited at 380 °C through chemical vapor deposition (CVD) as the GI. A 40-nm-thick *a*-IGZO layer was then deposited at 100 °C on the SiO_x_ GI from a sintered IGZO ceramic target (Advanced Nano Products Co., Ltd.) through RF magnetron sputtering with a mixed gas ratio of Ar:O_2_ = 90:10. A 100 nm-thick SiO_x_ etch stopping layer was prepared by CVD on the active layer at 300 °C. The source and drain electrodes were formed using 150 nm thick Mo. Finally, the fabricated IGZO TFTs (W/L = 40/8 um) were annealed in ambient air at 350 °C for 2 h before electrical measurements.

### Measurement

To form position-based defect states on the channel layer, pulse-type high voltage at the drain side (HVDS) was applied (1 kHz frequency, 1% duty cycle, 1 μs pulse rising/fall time, +40 V drain turn on voltage, and turn off voltage of 0 V @ *V*_*g*_ = 0 V)^18^. After HVDS was applied, the current signals were measured using a Keithley 4200 semiconductor characterization system. Here, we define the sensing direction for the samples as follows: forward sensing is in the same voltage/current direction of the source/drain with applied HVDS, and reverse sensing is in the opposite voltage/current direction of the source/drain with stress. Owing to the importance of the current characteristics in the driving IC chipset, we considered the physical properties of both directions with such a stress.

### TCAD Simulation

The device simulator TCAD was utilized in this study to understand electron transport properties. The simulation was conducted using Silvaco’s 2D ATLAS simulator package^22^. A configuration similar to that of fabricated materials and devices was employed. For the simulation, the following characteristic parameters were used as the input values. The relative permittivity of the silicon oxide as the gate insulator and *a*-IGZO as the channel semiconductor were 3.9 and 13, respectively; the electron affinity of *a*-IGZO was 4.1 eV; the band gap and mobility were 3.1 eV and 10 cm^2^V^−1^s^−1^, respectively; the effective density of the states in the conduction and valence bands were 5.0 × 10^18^ cm^−3^ and 4.6 × 10^19^ cm^−3^, respectively; and a fixed band-mobility model of 12 cm^2^ V^−1^s^−1^ was used. Table 1 summarizes the physical parameters employed in this study.

**Table 1:** Physical parameters for the TCAD Atlas 2D simulation.

Symbol	Description	Value
NTA	Density of acceptor-like tail state, A-T, at EC [cm^−3^ eV^−1^]	1.00E + 19
WTA	Slope of acceptor-like tail state, A-T [eV]	0.008
NGA	Peak density of acceptor-like Gaussian state, A-G [cm^−3^ eV^−1^]	1.50E + 17
EGA	Energy with peak density of acceptor-like Gaussian state, A-G [eV]	0
WGA	Slope of acceptor-like Gaussian state, A-G [eV]	0.64
NTD	Density of donor-like tail state, D-T, at EC [cm^−3^ eV^−1^]	3.00E + 19
WTD	Slope of donor-like tail state, D-T [eV]	0.1
NGD	Peak density of donor-like Gaussian state, D-G [cm^−3^ eV^−1^]	1.30E + 17
EGD	Energy with peak density of donor-like Gaussian state, D-G [eV]	2.48
WGD	Slope of acceptor-like Gaussian state, D-G [eV]	0.24

### Data availability

The datasets generated during and/or analyzed during the current study are available from the corresponding author on reasonable request.

## References

[1] Nomura K (2004). Room-temperature fabrication of transparent flexible thin-film transistors using amorphous oxide semiconductors. Nature.

[2] Toshio K, Kenji N, Hideo H (2010). Present status of amorphous In–Ga–Zn–O thin-film transistors. Science and Technology of Advanced Materials.

[3] Fortunato E, Barquinha P, Martins R (2012). Oxide semiconductor thin-film transistors: a review of recent advances. Advanced Materials.

[4] Hays DC, Gila BP, Pearton SJ, Ren F (2017). Energy band offsets of dielectrics on InGaZnO4. Applied Physics Reviews.

[5] Lany S, Zunger A (2005). Anion vacancies as a source of persistent photoconductivity in II-VI and chalcopyrite semiconductors. Physical Review B.

[6] Janotti A, Van de Walle CG (2007). Native point defects in ZnO. Physical Review B.

[7] Lany S, Zunger A (2010). Many-body GW calculation of the oxygen vacancy in ZnO. Physical Review B.

[8] Yeon H-W (2016). Structural-relaxation-driven electron doping of amorphous oxide semiconductors by increasing the concentration of oxygen vacancies in shallow-donor states. NPG Asia Mater.

[9] Van de Walle CG (2000). Hydrogen as a Cause of Doping in Zinc Oxide. Physical Review Letters.

[10] Nomura K, Kamiya T, Ohta H, Hirano M, Hosono H (2008). Defect passivation and homogenization of amorphous oxide thin-film transistor by wet O2 annealing. Appl. Phys. Lett..

[11] Wang XJ, Buyanova IA, Chen WM, Pan CJ, Tu CW (2008). Effects of stoichiometry on defect formation in ZnO epilayers grown by molecular-beam epitaxy: An optically detected magnetic resonance study. Journal of Applied Physics.

[12] Yu X, Marks TJ, Facchetti A (2016). Metal oxides for optoelectronic applications. Nat Mater.

[13] Lee H (2016). Electric Field-aided Selective Activation for Indium-Gallium-Zinc-Oxide Thin Film Transistors. Scientific Reports.

[14] Tak YJ (2016). Activation of sputter-processed indium–gallium–zinc oxide films by simultaneous ultraviolet and thermal treatments. Scientific Reports.

[15] Anderson J, Chris GVDW (2009). Fundamentals of zinc oxide as a semiconductor. Reports on Progress in Physics.

[16] Nomura K, Kamiya T, Hirano M, Hosono H (2009). Origins of threshold voltage shifts in room-temperature deposited and annealed a-In–Ga–Zn–O thin-film transistors. Appl. Phys. Lett..

[17] Chowdhury MDH, Migliorato P, Jang J (2011). Time-temperature dependence of positive gate bias stress and recovery in amorphous indium-gallium-zinc-oxide thin-film-transistors. Appl. Phys. Lett..

[18] Lee H-J, Cho SH, Abe K, Lee M-J, Jung M (2017). Impact of transient currents caused by alternating drain stress in oxide semiconductors. Scientific Reports.

[19] Urakawa S (2013). Thermal analysis of amorphous oxide thin-film transistor degraded by combination of joule heating and hot carrier effect. Appl. Phys. Lett..

[20] Uraoka Y, Hirai N, Yano H, Hatayama T, Fuyuki T (2004). Hot carrier analysis in low-temperature poly-Si TFTs using picosecond emission microscope. IEEE Transactions on Electron Devices.

[21] Tai Y-H, Huang S-C, Lin CW, Chiu HL (2007). Degradation of the Capacitance-Voltage Behaviors of the Low-Temperature Polysilicon TFTs under DC Stress. Journal of The Electrochemical Society.

[22] ATLAS Device Simulation Software User’s Manual. *Silvaco International*, Santa Clara, CA (2015).

